# Indoor air bacterial and fungal burden in the environment of an atopic child: implications for elevated urine mycotoxin levels

**DOI:** 10.2478/aiht-2026-77-4024

**Published:** 2026-03-30

**Authors:** Ivona Majić, Adela Krivohlavek, Elvira Kovač Andrić, Ranka Godec

**Affiliations:** Andrija Štampar Teaching Institute of Public Health, Division of Sanitary Microbiology, Zagreb, Croatia; Andrija Štampar Teaching Institute of Public Health, Food Safety and Quality Centre, Zagreb, Croatia; Josip Juraj Strossmayer University of Osijek, Osijek, Croatia; Institute for Medical Research and Occupational Health, Division of Environmental Hygiene, Zagreb, Croatia

**Keywords:** children’s health, indoor spaces, mesophilic bacteria, moulds, poor sanitary conditions, mezofilne bakterije, neadekvatni sanitarni uvjeti, plijesni, unutarnji prostori, zdravlje djece

## Abstract

Here we present a case of an atopic boy from Zagreb, Croatia, whose elevated urine mycotoxin levels prompted us investigate whether they were associated with microbial indoor air burden in the child’s primary and music school and family home. The music school had been water-damaged / mould-infested but had been repaired by the time of our measurements. We also measured urine mycotoxin in one healthy child who attended the same elementary school and in all household members of the atopic boy. The results showed no microbial contamination at any of the measurement locations, with bacterial concentrations ranging from 172 to over 570 CFU/m^3^ of air and fungal concentrations between 67 and 82 CFU/m^3^, all determined only in the music school classroom. The dominant species isolated in the music school were *Aspergillus fumigatus* (55 CFU/m^3^) and *Penicillium verrucosum* (24 CFU/m^3^), both potential mycotoxin producers. Considering that only the atopic boy had elevated urine mycotoxin levels, we believe that mould in the music school cannot be ruled out as the source of exposure. In fact, we believe our case illustrates that safety thresholds for mould exposure can vary and may be much lower for immunocompromised or sensitive individuals.

## Abstract (hr)

Ovdje prikazujemo studiju slučaja atopičnog dječaka iz Zagreba, Hrvatska, čije su povišene razine mikotoksina u urinu potaknule ispitivanje njihove moguće povezanosti s mikrobnim opterećenjem zraka u zatvorenim prostorima njegove osnovne i glazbene škole te obiteljskog doma. Zgrada glazbene škole prethodno je bila oštećena vodom i zahvaćena plijesni, no do vremena naših mjerenja bila je sanirana. Također smo izmjerili mikotoksine u urinu jednog zdravog djeteta koje je pohađalo istu osnovnu školu te svih članova kućanstva atopičnog dječaka. Rezultati nisu pokazali mikrobnu kontaminaciju ni na jednoj od mjernih lokacija, pri čemu su se bakterijske koncentracije kretale od 172 do više od 570 CFU/m^3^ zraka, a gljivične između 67 i 82 CFU/m^3^, i to isključivo u učionici glazbene škole. Dominantne izolirane vrste u glazbenoj školi bile su *Aspergillus fumigatus* (55 CFU/m^3^) i *Penicillium verrucosum* (24 CFU/m^3^), obje potencijalni proizvođači mikotoksina. S obzirom na to da je samo atopični dječak imao povišene razine mikotoksina u urinu, smatramo da se plijesan u glazbenoj školi ne može isključiti kao izvor izloženosti. Štoviše, smatramo da naš prikaz slučaja upućuje na to da sigurnosni pragovi izloženosti plijesni mogu varirati te biti znatno niži kod imunokompromitiranih ili osjetljivih osoba.

People typically spend most of their time indoors (around 80–95 %), where they breathe in 10–14 m^3^ of air a day (1,2,3,4). The quality of indoor air significantly influences human health, and the World Health Organization (WHO) has expressed concern about the negative health impacts of humidity in buildings and exposure to airborne bacteria and fungi, as they can cause respiratory issues, allergies, asthma, and immune system disorders (5,6,7). Poor ventilation and thermal insulation favour the growth of microorganisms indoors and present a risk for vulnerable population groups, such as children and the elderly (8,9,10,11,12,13).

Of particular concern are moulds producing mycotoxins, capable of affecting many organs, including the immune and nervous systems, lungs, kidneys, and liver, especially in sensitive individuals (e.g. immunocompromised and/or genetically predisposed to slow elimination) (14,15,16,17). Yet, regulatory agencies like the United States Environmental Protection Agency and Food and Drug Administration have not set safe exposure standards for indoor moulds (18). Chronic exposure to mycotoxins has been reported to cause fatigue, nausea, immunotoxicity, neurotoxicity (e.g. dizziness, anxiety, depression, and cognitive deficits), pulmotoxicity, nephrotoxicity, hepatotoxicity, birth defects, and cancer (19,20,21,22,23). Furthermore, exposure to mycotoxins can make individuals vulnerable to microbial diseases (24). In a recent survey by the Australian government, tenants living in mouldy apartments reported biotoxin-related symptoms (25), which drew attention to the dangers of under-investigated airborne mycotoxins.

Air quality in school buildings may be even more problematic because of greater occupancy and poor ventilation and maintenance, leading to higher absenteeism in susceptible children (26, 27). These issues are even more pronounced in music schools, as they also involve activities favouring microbial dispersion via playing instruments and singing (28, 29).

Considering that the presence of airborne bacteria and fungi and potential mycotoxin exposure has hardly been addressed in children in Croatia, our aim was to get a comprehensive view based on the experience of one atopic 12-year-old boy by measuring exposure in indoor environments in which he spends most of his time and see if there was a link between mycotoxin exposure and atopy. The specific goals were: 1) to quantify air bacterial and fungal levels in all three environments; 2) identify fungal species; and 3) investigate potential associations between the mycotoxins detected in the urine of the atopic boy and airborne fungi found in the three investigated environments.

## MATERIALS AND METHODS

### Case areas

Air samples were taken from the boy’s primary school, music school classroom, and a family home in March 2024.

The music school accommodates 20 students a day and consists of a classroom that used to be a nuclear bunker in the past (therefore with no windows). Humidity in there was controlled with an air dehumidifier working the whole time, and an air-conditioning device, which was turned off during air sampling. Otherwise, the device had been used for both heating and cooling. No mould was visible, but the room had a specific mouldy scent that remained from a pipe leakage that occurred during the summer of 2023, when the school was closed.

The primary school accommodates around 660 children a day. To assess air quality and microclimatic conditions, air samples were collected from four locations, namely the boy’s classroom, the gym, the locker room, and the central lobby to establish potential variations between them. The primary school was centrally heated during the measurements.

The boy’s residence is a two-bedroom flat on the second floor (level three) of a ten-year-old multi-storey building in Novi Zagreb, which is part of the Croatia’s capital’s densely populated metropolitan area. The flat has four permanent residents and is a representative urban living space. Bioaerosol samples were collected and microclimate conditions measured in the bathroom, bedroom, and the living room to establish potential differences in microbial presence related to occupancy, moisture, and ventilation. The flat was centrally heated during the measurements.

### Air sampling and microorganism counting

Air samples were collected with a SAS Super DUO 360 air sampler (VWR International, Radnor, PA, USA) at the 180 L/min rate to obtain two different air volumes in parallel (2×100 L and 2×200 L) for bacterial counts and two different air volumes in parallel (2×100 L and 2×200 L) for mould counts for each measurement location. Four dichloran-glycerol 18 % (DG18) agar plates and four Tryptone soy agar (TSA) plates were used for each sampling point, all obtained from Oxoid Ltd. (Basingstoke, UK).

The sampler operates by drawing air through a perforated lid (401 holes) and directing it onto a 90 mm Petri dish containing either TSA agar for the cultures of aerobic mesophilic bacteria or DG18 agar for fungal cultures. Both indoor and outdoor air sampling followed the respective ISO norms (29,30,31,32,33,34,35,36).

After sampling, the TSA dish was incubated at (37±3) °C for two days. If there was no colony growth, incubation was extended to up to seven days. The DG18 plate for determining fungal (mould) count was incubated at (25±3) °C for seven days, while the incubation for fungal identification lasted 10 days.

According to the ISO standards and instruction manual (29,30,31,32,33,34,35,36,37), the number of counted mould colonies on the agar does not need to be adjusted, while the number of bacteria does. Mould concentration per cubic metre of air is obtained by calculating the number of colonies for each identified species individually. Unidentified species are categorised as “others”.

The number of bacteria counted on the surface of the TSA plates must first be corrected for the statistical possibility of multiple particles passing through the same hole. Correction tables are given in the manual (37). The probable count (*P_r_*) is then used to calculate the colony forming units (CFU) per cubic metre of sampled air.

The total mould colony count is the sum of identified and unidentified mould species and is calculated according to the following equation (Eq. 1):

[1] $${\text{C}}_{\text{i}}={\text{n}}_{\text{CFU}}/{\text{V}}_{\text{i}}$$

where *V_i_* is the volume of sampled air, *n_CFU_* the number of CFU, and *C_i_* the number of CFU per m^3^ of air.

The total bacterial colony count was calculated according to the following conversion formula taken from the SAS device user’s manual (37) (Eq. 2):

[2], $$\text{X}=\frac{{\text{P}}_{\text{r}}\times 1000}{\text{V}}$$

where *V* is the volume of sampled air, *P_r_* probable number of CFU obtained from the correction table, and *X* the number of CFU per m^3^ of air.

### Identification of fungi

Fungal isolates incubated on DG18 plates for 3–5 days were identified using the matrix-assisted laser desorption/ionisation time-of-flight mass spectrometry (MALDI-TOF MS) (MALDI Biotyper microflex LT/SH, Bruker, Billerica, MA, USA) as described in detail elsewhere (38). For each sample we used two 1.5 mL tubes containing 500 µL of 100 % ethanol and 50 µL of 0.1 mm diameter silica beads. Then we added spores (conidia) and hyphae taken with a swab from a 1–2 cm diameter circle of mould in the agar plate. The collected material was suspended and centrifuged at 14,000 *g* for 3 min. The ethanol supernatant was removed, and the pellet resuspended in 20 µL of 70 % formic acid. After a brief vortexing, 20 µL of acetonitrile was added, vortexed, and centrifuged again at 14,000 *g* for 3 min. 1 µL of supernatant was added and applied in a reusable 96-well stainless steel plate, while 1 µL of Bruker bacterial test standard (BTS, *E. coli* spike with two high molecular weight proteins) suspension was added to two previously marked wells for calibration. All wells were air-dried, overcoated with 1 µL of α-cyano-4-hydroxycinnamic acid (HCCA) matrix, air-dried for another 2 min, and placed in the MALDI Biotyper for analysis in positive ion mode, which detects protonated peptides and proteins from the sample.

### Urine sampling and mycotoxin measurement

One urine sample was collected in the morning from the atopic child, and one from a random healthy child who did not attend the music school. Urine samples were also taken from all household members living with the atopic child. The urine collection cups were placed frozen and placed in a clear ziplock bag for biohazardous materials, which was put in a silver thermal bag and an absorbent packing sheet and shipped to a Mosaic Diagnostics laboratory (Overland Park, KS, USA) following the established lab protocol (39).

Mycotoxins were determined with the MycoTOX Profile (Mosaic Diagnostics), a modern test which utilises liquid chromatography to detect up to 11 free (unconjugated) mycotoxins even at low levels as described elsewhere (39).

## RESULTS AND DISCUSSION

Table 1 shows the microclimatic conditions at the sampling locations measured in the spring of 2024.

**Table 1:** Microclimatic conditions at the sampling locations

	**Air temperature (°C)**	**Relative humidity (%)**	**No. of people present**
**Music school**			
Entrance	15.6	41.2	3
Middle area	15.8	41.5	3
Rear area	15.2	40.7	3
Outdoor air	12.7	38	2
**Primary school**			
Classroom	22.4	45.3	19
Sports hall	21.5	46.5	7
Gym locker room	22.2	41.4	1
Lobby	22.7	38.4	22
Outdoor air	15.3	44	2
**Apartment**			
Bathroom	23.5	42.3	1
Bedroom	21.2	46.4	1
Living room	23.2	43.4	5
Outdoor air	15.6	44	2

**Figure 1:**
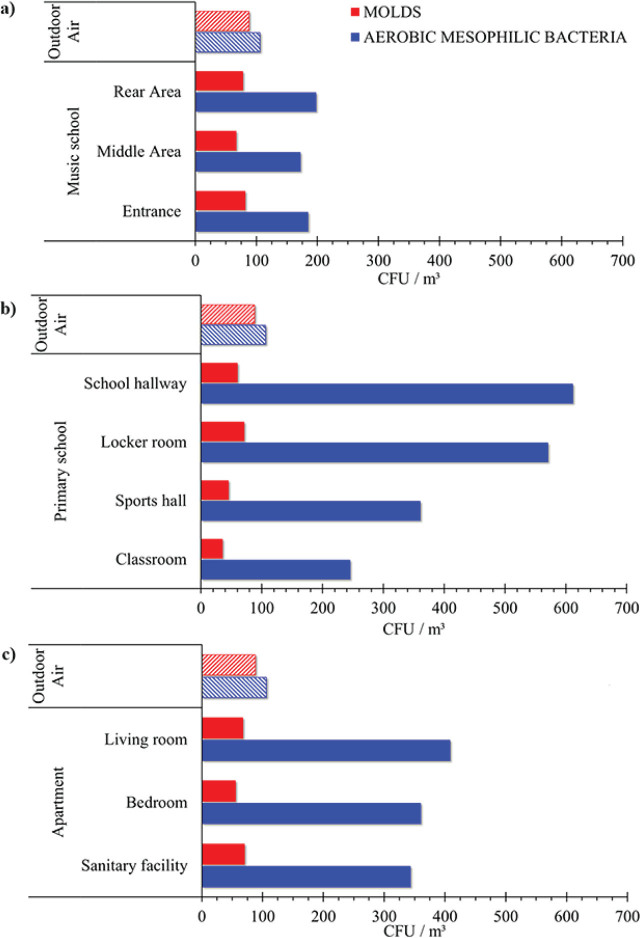
Total fungal (red) and bacterial (blue) counts in different areas of the a) music school, b) primary school, and c) apartment, and the respective outdoor counts

Figure 1 shows the indoor air bacterial and fungal loads across all three investigated locations. The highest bacterial loads were measured in the primary school (245–570 CFU/m^3^), followed by the flat (343–408 CFU/m^3^), while the lowest load was measured in the music school classroom (172–198 CFU/m^3^). Although there are no generally accepted limits for bacterial concentrations in indoor air, the WHO recommends that the total microbial concentration should not exceed 1000 CFU/m^3^ (1, 2), while others recommend the limit of 750 CFU/m^3^ (40). According to the European Commission’s sanitary standards for non-industrial premises (41), the permissible limits for bacterial loads are ≤500 CFU/m^3^. Since the variation in indoor bacterial loads is mainly owed to external environmental factors, our comparison suggests that all indoor loads were within the acceptable limit according to the Portuguese ordinance No. 353-A/2013 (42), which recommends that indoor bacterial concentrations should not exceed outdoor concentrations for more than 350 CFU/m^3^.

The highest fungal load was recorded in the music school classroom, more precisely at its entrance (82 CFU/m^3^) and in the back (78 CFU/m^3^), and the lowest was in middle area (67 CFU/m^3^) (Figure 1), yet it did not surpass the outdoor measurements (88 CFU/m^3^).

MALDI-TOF analysis identified an impressive diversity of 55 mould and one yeast species, the most frequent of which are listed in Table 2. The dominant species isolated in the music school sample was *Aspergillus fumigatus* (55 CFU/m^3^), which produces some of the most significant mycotoxins, including aflatoxin, gliotoxin, and ochratoxin A (43,44,45). *A. fumigatus* is common in mouldy buildings, and the gliotoxin or analogue-producing isolates have been reported on wood, plasterboard, and chipboard (46). Another potential mycotoxin producer, found only in the music school classroom, was *Penicillium verrucosum*. *Alternaria alternata* was found in all locations, and even though it does not produce mycotoxins, it is a very common cause of allergic reactions and asthma in sensitive people (47). Its concentrations were higher in the flat (40 CFU/m^3^) and primary school (42 CFU/m^3^) than in the music school (10 CFU/m^3^).

**Table 2:** Types of isolated moulds from indoor air samples taken at the three locations by investigated area

**Location**	**Identified mould isolates**
**Music school**	

Entrance	*Aspergillus flavus* (11)
	*Penicillium* spp. (6)
	*Aspergillus fumigatus* (29)
	*Candida albicans* (3)

Middle area	*Alternaria alternata* (5)
	*Penicillium verrucosum* (12)
	*Aspergillus fumigatus* (13)
	*Aspergillus flavus* (10)

Rear area	*Penicillium* spp. (7)
	*Penicillium verrucosum* (12)
	*Aspergillus fumigatus* (13)
	*Aspergillus flavus* (10)
	*Alternaria alternata* (5)

**Primary school**	

Classroom	*Alternaria alternata* (8)
	*Aspergillus flavus* (3)
	*Candida albicans* (2)
	Other (2)

Sports hall	*Aspergillus niger* (13)
	*Alternaria alternata* (11)
	*Wallemia* spp. (3)

Gym locker room	*Penicillium* spp. (5)
	*Alternaria alternata* (10)
	Other (3)

Lobby	*Alternaria alternata* (14)
	*Aspergillus niger* (15)
	*Penicillium* spp. (4)
	Other (3)

**Apartment**	

Bathroom	*Alternaria alternata* (15)
	*Aspergillus flavus* (3)
	*Candida albicans* (12)
	*Paecilomyces variotii* (18)

Bedroom	*Aspergillus niger* (13)
	*Alternaria alternata* (11)
	*Paecilomyces variotii* (6)
	Other (3)

Living room	*Paecilomyces variotii* (9)
	*Alternaria alternata* (14)
	*Aspergillus niger* (13)
	Other (4)

Considering the Portuguese ordinance No. 353-A/2013 (42) our mould levels are all within the reference ranges (indoor air mould concentrations must be lower than he outdoor ones), which suggests that the fungal burden at all three premises is safe for health.

However, it is important to note that mycotoxins and other secondary metabolites are released from fungal spores and colony fragments after inhalation and that even low concentrations relative to outdoor air may produce a toxic effect (48,49,50,51), especially in low humidity environments, which stimulate release of spores, which are even more dispersed in the air through indoor physical activity such as that pertinent to music schools (52). We therefore find a link between moulds isolated in the music school and mycotoxins isolated in the urine of our atopic child quite possible.

**Figure 2:**
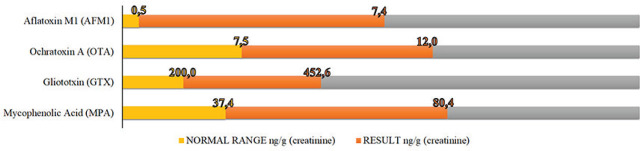
Mycotoxin levels (ng per g of creatinine) isolated from the child’s morning urine sample

Speaking of the latter, Figure 2 shows elevated mycotoxin levels in the child’s urine, namely aflatoxin M1 (7.4 ng/g of creatinine), ochratoxin A (12 ng/g of creatinine), gliotoxin (452.6 ng/g of creatinine), and mycophenolic acid (80.4 ng/g of creatinine), while the respective normal thresholds are 0.5, 7.5, 200, and 37.4 ng/g of creatinine. Similar elevated mycotoxin concentrations were reported in residents of moisture-damaged and mould-infested buildings (18, 49, 53,54,55,56,57,58,59). In contrast, mycotoxin levels in control urine samples (those taken from a healthy pupil and household member of the atopic child) were below the detection limits.

Recent studies (49, 60) indicate that various indoor mould species trigger strong inflammatory and allergic reactions well fungal rhinosinusitis in asthmatics sensitised to moulds. There is valid epidemiological evidence that atopic people (those genetically predisposed to allergies and asthma) are sensitive to mould spores and that moulds contribute to upper respiratory health problems in children (51).

Even though the indoor air levels measured in our study are within the Portuguese reference range, we found yeasts and moulds that could adversely affect health, particularly in individuals with compromised immune systems (60,61,62,63). One of the worst-case scenarios involves water intrusion, such as that reported for the music school (former nuclear bunker) in our study, which favours the formation of large amounts of biomass and mycotoxins, followed by a drying period that favours the spread of spores and colony fragments and their indoor deposition (64). Mycotoxins can persist in indoor environments for long, even after renovation/restoration. Therefore, it remains possible that the source of the elevated urine mycotoxins in the child was the mould present in the air of the music school classroom, particularly given that no water intrusions were reported in either the primary school or in the child’s flat.

### Study limitations

Our study is limited to only one atopic child and involves onetime measurements (no follow-up). As result, we could not be establish a clear association between exposure and effect or the absence thereof.

## CONCLUSIONS

Although we assumed that indoor measurements and microbiological analyses would confirm a clear association between the measured mycotoxin level in the urine of atopic child and impaired indoor air quality, this was not the case. A possible reason arises from the short total period of monitoring and sampling that reflects the current state in the environment in which the child was staying for classes shortly before all analyses were conducted.

Nevertheless, the presence of mycotoxins in the child’s urine, which was not established in either the other child or the child’s relatives, cannot exclude the risk of exposure to mycotoxin producers in the music school, which should further be investigated in a larger number of participants over a longer period of time.
